# Single Whole-Body Cryostimulation Procedure versus Single Dry Sauna Bath: Comparison of Oxidative Impact on Healthy Male Volunteers

**DOI:** 10.1155/2015/406353

**Published:** 2015-03-19

**Authors:** Paweł Sutkowy, Alina Woźniak, Paweł Rajewski

**Affiliations:** ^1^The Chair of Medical Biology, Nicolaus Copernicus University, Ludwik Rydygier Collegium Medicum in Bydgoszcz, Karlowicza 24, 85-092 Bydgoszcz, Poland; ^2^The Department of Nephrology and Internal Medicine, City Hospital in Bydgoszcz, Szpitalna 19, 85-826 Bydgoszcz, Poland

## Abstract

Exposure to extreme heat and cold is one of the environmental factors whose action is precisely based on the mechanisms involving free radicals. Fluctuations in ambient temperature are among the agents that toughen the human organism. The goal of the study was to evaluate the impact of extremely high (dry sauna, DS) and low (whole-body cryostimulation, WBC) environmental temperatures on the oxidant-antioxidant equilibrium in the blood of healthy male subjects. The subjects performed a single DS bath (*n* = 10; 26.2 ± 4.6 years) and a single WBC procedure (*n* = 15; 27.5 ± 3.1 years). In the subjects' blood taken immediately before and 20 min after the interventions, the activity of superoxide dismutase (SOD), catalase (CAT), and glutathione peroxidase (GPx) and the concentration of thiobarbituric acid reactive substances in erythrocytes (TBARSer) and blood plasma (TBARSpl) were determined. Single WBC and DS procedures induced an increase in the activity of SOD and GPx, as well as SOD and CAT, respectively. The SOD activity was higher after WBC than after DS. Extremely high and low temperatures probably induce the formation of reactive oxygen species in the organisms of healthy men and, therefore, disturb the oxidant-antioxidant balance.

## 1. Introduction

All aerobic organisms constantly resist and counteract the harmful effects of reactive oxygen species (ROS). Free radicals including oxygen free radicals (OFR) are by-products of incomplete reduction of oxygen molecule [1]. They are produced in organisms either as by-products of metabolism or as a response to the action of external agents such as environmental factors. The factors may act in different ways but the main mechanism of their action is related with an increased generation of ROS. As regards biological aspects, stress reaction induced by ROS is manifested by structural and chemical changes of organic substances and, in consequence, disorders of tissue metabolism. It may also trigger adaptive mechanisms, including improvements in the antioxidant capacity [2]. Aerobic organisms have developed a variety of mechanisms of antioxidant defence. The mechanisms protect the integrity of the organisms against excessive concentrations of ROS, that is, in the course of oxidative stress. Thus, the antioxidant defence system is responsible for the protection against damage caused by free radicals. Any substance that defends a substrate from oxidative damage is called an antioxidant. Undoubtedly, superoxide dismutase (SOD), glutathione peroxidase (GPx), and catalase (CAT) are the enzymes that play a major role in the enzymatic scavenging of ROS. In addition to the antioxidant enzymes, nonenzymatic scavengers, present in cell membranes and extracellular fluids, play an equally important role. Among the nonenzymatic free radical scavengers are proteins, albumins, and the so-called low-molecular weight antioxidants, such as ascorbic acid, glutathione, and *α*-tocopherol. Thus, the occurrence of oxidative stress is a sign of inefficiency of the antioxidant defence system [3].

Temperature is one of the environmental factors that causes an increased generation of ROS in the human organism. Both very low and very high temperatures are able to temper human body. Tempering is precisely based on the mechanisms involving free radicals [4–7]. One of the methods that use this low-temperature effect is whole-body cryostimulation (WBC). The method involves remaining for 1–3 min in the so-called cryo-chamber at an extremely low temperature (from −160°C to −120°C) [8]. The stimulation, combined with intense kinesiotherapy, has special recognition in the treatment of inflammatory arthritis syndromes (i.e., ankylosing spondylitis, AS, and rheumatoid arthritis, RA), fibromyalgia, multiple sclerosis, osteoporosis, overweight, and obesity, as well as in treating sport injuries and in postexercise recovery [7]. The other method, sauna bath, is a curing procedure involving surface application of extremely high temperature in an insulated wooden room, heated to between +45°C and +120°C (depending on the type of sauna), for 5–20 min. The procedure is repeated one to three times for better results. Sauna baths are often used as a supplementary treatment in patients suffering from cardiovascular diseases, depression, and respiratory diseases, especially chronic obstructive pulmonary disease (COPD), as well as from diseases of the musculoskeletal system (usually in fibromyalgia, but also in RA) [9, 10]. This method is also a source of ROS [6, 11]. Nonetheless, sauna is predominantly used for wellness and physical recovery after workout [12, 13]. Currently, the most popular type of sauna is the Finnish sauna, also called dry sauna (DS) [9].

The aim of the study was to investigate the effect of single WBC and DS sessions on the oxidant-antioxidant equilibrium in healthy men who volunteered for the study. In the subjects' blood, the activity of CAT, SOD, and GPx and the concentration of thiobarbituric acid reactive substances (TBARS) were determined.

## 2. Material and Methods

### 2.1. Participants

Healthy men, volunteers, performed a single DS bath (*n* = 10) and a single WBC procedure (*n* = 15). The subjects' average age was 26.2 ± 4.6 years in the DS group and 27.5 ± 3.1 years in the WBC group. All the men who volunteered for the study had never used sauna bathing or cryostimulation before the study period. No subject suffered from obesity or cardiovascular or pulmonary diseases. Every subject exercised moderate physical activity and their diet was varied with no supplementation. Additional features of the subjects are shown in Table 1. Moreover, the subjects were informed about the goal of the study and signed the consent forms. The study was approved by the Bioethics Committee at Nicolaus Copernicus University, Ludwik Rydygier Collegium Medicum in Bydgoszcz (nos. KB189/2012 and KB657/2012).

### 2.2. Study Design

The single WBC treatment (−120°C, 2 min) was carried out at the Olympic Preparation Centre (OPC) in Walcz, Poland. The subjects who entered the cryo-chamber wore shorts, socks, gloves, and headbands for ear protection. Wooden clogs were used as a protection from foot frostbites, whereas the lungs were protected by a surgical face mask (with “double gauze pad layer”). The subjects were informed about the contraindications of WBC (hypertension, circulatory failure) and the necessity of taking inhalations two times shorter than exhalations.

The single DS bath was performed at the Sport Factory Fitness Centre, Bydgoszcz, Poland, at +90°C, 10% of relative air humidity, for 40 minutes. The DS bath was divided into 3 consecutive exposures. Each of these exposures lasted for 10 minutes plus approximately 10 minutes for cooling the body with a cold shower and a rehydration between the trials. The subjects entered the sauna in pool slippers.

Blood taken from the basilic vein into test tubes before and 20 min after the DS or WBC sessions was subsequently used for the assays of selected oxidative stress parameters.

### 2.3. TBARS Measurement

The thiobarbituric acid reactive substances were determined in both the subjects' plasma (TBARSpl) and erythrocytes (TBARSer) using the method described by Buege and Aust [14] and modified by Esterbauer and Cheeseman [15]. TBARS, as lipid peroxidation products containing mainly malondialdehyde (MDA), were identified using thiobarbituric acid (TBA). To the hemolysate or 0.5 mL of plasma, 4.5 mL of reactive mixture containing 0.375% of TBA and 15% of trichloroacetic acid (TCA) in 0.25 M HCl was added. In order to prevent the formation of lipid peroxidation products, 0.01% solution of 3.5-diisobutyl-4-hydroxytoluene (BHT), an inhibitor of lipid peroxidation, was added into the test tubes during the reaction. The samples were incubated for 20 minutes in hot water (+100°C), cooled down, and centrifuged at 2000 ×g for 15 minutes at +4°C. The absorbance of the samples (supernatant) was measured at the wavelength of 532 nm. The TBARSer concentration was expressed in nmol MDA per g of haemoglobin and the TBARSpl was expressed in nmol MDA per mL of blood plasma.

### 2.4. SOD, CAT, and GPx Measurement

The activity of antioxidant enzymes was determined in the subjects' erythrocytes. The CAT activity was determined using the Beers and Sizer method [16]. The method is based on a decrease in the absorbance of a hydrogen peroxide (H_2_O_2_) solution. H_2_O_2_ is decomposed by the enzyme, so the decrease in the absorbance is directly proportional to its activity. The CAT activity was expressed in 10^4^ IU per g of haemoglobin.

The GPx activity was determined according to the Paglia and Valentine method [17]. The principle of the method is based on the decomposition of H_2_O_2_ by GPx with the simultaneous oxidation of reduced glutathione. Oxidized glutathione is then reduced in a reaction catalysed by glutathione reductase. The role of a coenzyme in this reaction is played by reduced nicotinamide adenine dinucleotide phosphate (NADPH), which is converted into an oxidized form and induces a change in the absorbance of the light. The GPx activity was expressed in U per g of haemoglobin.

The SOD activity was assayed using the method by Misra and Fridovich [18]. The method is based on an inhibition of adrenaline oxidation to adrenochrome in alkaline environment, which induces a change in the extinction of the solution. The activity of SOD was expressed in U per g of haemoglobin.

### 2.5. Statistical Analysis

The obtained results are presented as means ± standard deviations. Differences between means were tested using the Student's *t*-test for dependent and independent variables. Before running the *t*-test, the model assumptions were also verified using the Kolmogorov-Smirnov test for normality and the *χ* ^2^-test to assess the equinumerosity of the groups. Differences at a significance level of *P* < 0.05 were assumed as statistically significant.

## 3. Results

**Figure 1:**
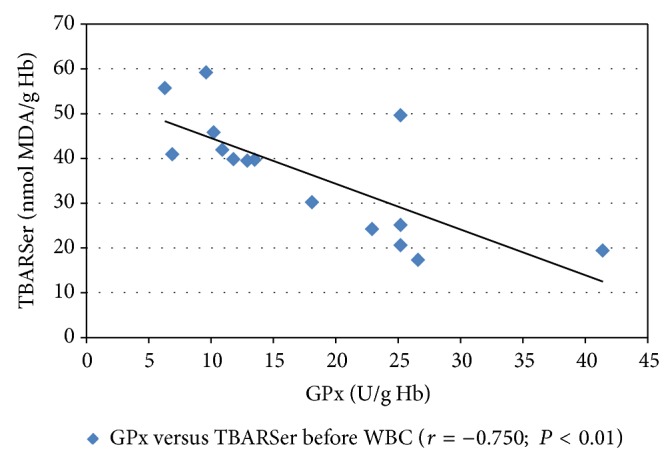
Pearson's correlation coefficient (*r*) between the TBARSer concentration and the GPx activity in the erythrocytes of healthy male subjects directly before the whole-body cryostimulation (WBC) treatment. TBARSer, thiobarbituric acid reactive substances in erythrocytes; GPx, glutathione peroxidase.

**Figure 2:**
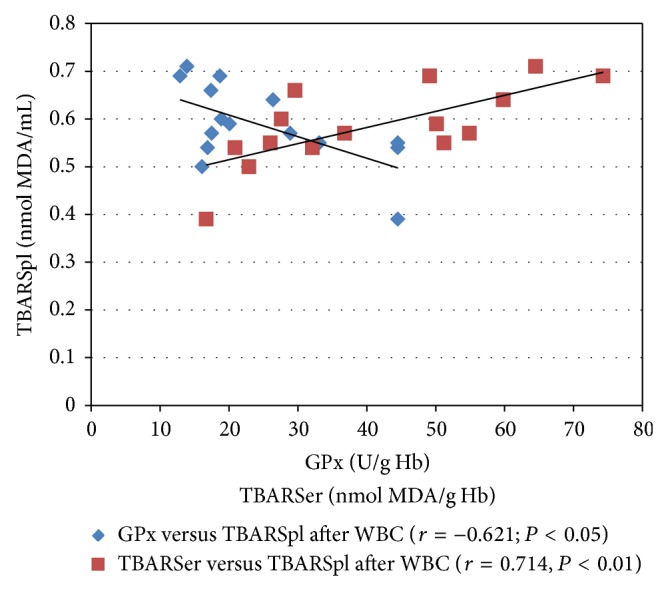
Pearson's correlation coefficient (*r*) between the concentrations of TBARSer and TBARSpl, as well as between the GPx activity and the TBARSpl concentration in the blood of healthy male subjects 20 min after the whole-body cryostimulation (WBC) treatment. TBARSer, thiobarbituric acid reactive substances in erythrocytes; TBARSpl, thiobarbituric acid reactive substances in blood plasma; GPx, erythrocytic glutathione peroxidase.

In the WBC group after the procedure, the SOD and GPx activities significantly increased (*P* < 0.001 and *P* < 0.01, resp.). The increase in the SOD activity in the subjects' erythrocytes after the WBC procedure amounted to 30.8% and the GPx activity amounted to 40.4% (Table 2). Furthermore, a negative Pearson's correlation coefficient (*r*) between the GPx activity and the TBARSer concentration after the WBC procedure was revealed (Figure 1). Twenty minutes after the WBC procedure, a positive correlation coefficient between the concentrations of TBARSer and TBARSpl and a negative correlation coefficient between the GPx activity and the TBARSpl concentration were found (*P* < 0.01 and *P* < 0.05, resp.; Figure 2).

**Figure 3:**
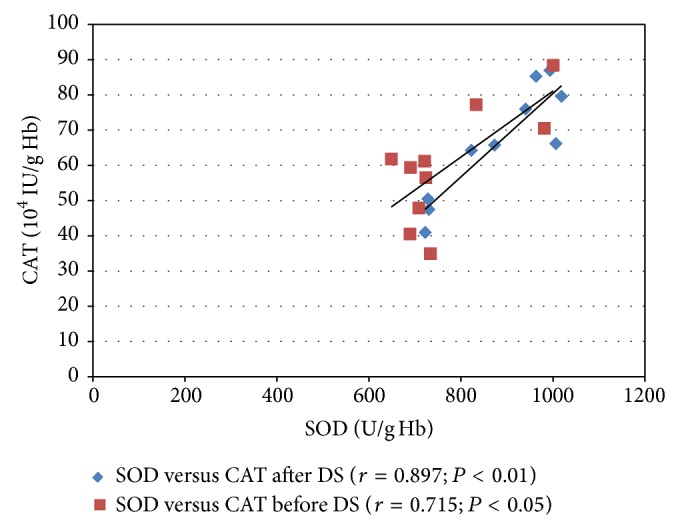
Pearson's correlation coefficient (*r*) between the activity of SOD and CAT in the erythrocytes of healthy male subjects directly before and 20 min after the dry sauna (DS) bath. SOD, superoxide dismutase; CAT, catalase.

In the DS group, the sauna bath induced a statistically significant increase in the SOD and CAT activities. After the DS procedure, the SOD activity increased by 13.8% (*P* < 0.05) and the CAT activity increased by 10.7% (*P* < 0.01; Table 2). Moreover, very high positive linear correlation coefficients between the activity of SOD and CAT, both directly before and 20 min after the DS bath, were observed (*P* < 0.05 and *P* < 0.01, resp.; Figure 3).

Both study groups differed in a statistically significant manner in oxidative stress parameters (Table 2). Before (baseline) and after the procedures, the TBARSpl, TBARSer, and GPx levels were higher in the participants subjected to WBC compared to the DS-treated subjects (*P* < 0.001). The SOD activity after the WBC procedure was higher by 17.5% compared to the activity measured after the DS bath (*P* < 0.01); however, the baseline values of the SOD activity did not differ (*P* > 0.05). There were no differences between the two groups with reference to the CAT activity (*P* > 0.05).

## 4. Discussion

The changes in the oxidative stress parameters in the WBC group reported in this paper are similar to the changes reported by Wozniak et al. [8] who studied 10 healthy men subjected to a single WBC procedure (−120°C for 3 min). The authors showed a lack of changes in the plasma and erythrocytic TBARS concentrations (*P* > 0.05) and an increase in the erythrocytic SOD (*P* < 0.001) and GPx (*P* < 0.01) activities 20 min after the procedure [8]. Similarly, no statistically significant differences in the TBARSpl and TBARSer concentrations were found in another study in the blood of 12 men immediately after a single WBC procedure (−120°C, 2 min) [5]. Moreover, the authors demonstrated that the plasma concentrations of vitamins A and E in the subjects did not change either (*P* > 0.05) [5]. Another study revealed a statistically significant decrease in the total oxidative status (TOS), but also in the total antioxidative status (TAS) 30 min after a single WBC procedure (−130°C, 3 min) in the blood of 15 clinically healthy men aged 21 [19]. Inconsistencies in the obtained data may probably be due to the reduced blood supply to deep tissues, which could be confirmed by the most recent findings regarding WBC by Selfe et al. [20]. The authors demonstrated a decrease in both deoxyhaemoglobin and oxyhaemoglobin for vastus lateralis a few minutes after the WBC procedure (−135°C, 1–3 min) in fourteen elite rugby players [20]. Moreover, Lubkowska et al. revealed no differences in TAS and TOS the day after the WBC procedure as compared with the initial values [19]. All the above mentioned data suggest that a single WBC procedure slightly disturbs the oxidant-antioxidant balance in healthy male subjects but is not sufficient to induce oxidative stress. Furthermore, in the paper, statistically significant high negative r-Pearson's correlations between the erythrocytic GPx activity and the TBARSer concentration before the WBC procedure, as well as between the GPx activity and the TBARSpl concentration after the procedure, were observed (Figures 1 and 2). The correlations, together with the changes in the SOD and GPx activities, as well as the TBARS concentrations, point additionally to the primary generation of ROS. Many papers confirm that WBC treatment has a prooxidative effect that results in adaptive changes [7, 8, 21–23]. Therefore, those references, along with the results of this paper, indicate that cryostimulation induces an increase in the production of ROS in humans which is insufficient to cause oxidative stress but sufficient to trigger beneficial antioxidant processes.

Dry sauna was found to have a similar effect. The increase in the SOD (*P* < 0.05) and CAT activities (*P* < 0.01) after a single DS bath reported in this paper proves the increase in the concentrations of superoxide anion (O_2_^∙−^) and hydrogen peroxide (H_2_O_2_) in the subjects' blood. Joint involvement of SOD and CAT in the scavenging of ROS was confirmed by the very high positive linear correlation coefficients (Figure 3). Sutkowy et al. [6] also found a statistically significant increase in the SOD activity induced by dry sauna bath. In seven healthy men exposed to an extremely high temperature (+90°C) three times for 10 minutes, the SOD activity increased both 15 and 60 minutes after the bath as compared with control (measurement performed directly before the bath; *P* < 0.05) [6]. A disturbance in the oxidant-antioxidant balance and adaptive changes after dry sauna bath in healthy young men were also investigated by Zinchuk and Zhadko [11]. After the sauna bath at the start of the experiment (*n* = 13), a statistically significant increase in the concentrations of conjugated dienes (CD), malondialdehyde (MDA), and Schiff bases in both the subjects' blood plasma and erythrocytes was observed, along with a simultaneous decrease in the erythrocytic CAT activity and the plasma concentration of vitamin E. At the end of the experiment (*n* = 11), an adaptive effect was observed: the men became accustomed to the extremely high temperature. After the sauna bath, a postexposure increase was observed only in the erythrocytic MDA and plasma Schiff bases concentrations, while a decrease was observed in the erythrocytic CAT activity. The authors also revealed an increase in the concentration of total nitrite after the sauna bath both at the start and at the end of the experiment. The authors explained the results by an increase in the nitric oxide (NO) concentration [11]. However, it is unclear why the results obtained by Zinchuk and Zhadko [11] are not consistent with normal distribution and what is meant by “the start of the course” and “the end of the course.” The authors only reported that the whole experiment consisted of 20 sauna baths once a week for 5 months. Each bath consisted of two exposures to +85–+90°C, 10–15% of relative air humidity, 5–10 min, followed by cooling down at room temperature (+20–+21°C) for 5 min. Other authors also take into account a stress effect of sauna baths in the context of increased ROS generation and the resulting antioxidant response and adaptation of the human organism [4, 24, 25].

To sum up, the literature describes sauna baths and whole-body cryostimulation as agents that disturb the oxidant-antioxidant balance slightly towards oxidation. Therefore, the effect allows a gradual adaptation and hardening of the organism in the long-term use. The presented study also revealed this effect, but, due to rather small study groups, further studies are necessary to confirm these findings.

## 5. Conclusions

A single whole-body cryostimulation procedure and a single dry sauna bath probably induce the formation of reactive oxygen species in the organisms of healthy men and, therefore, disturb the oxidant-antioxidant balance.

## Figures and Tables

**Table 1:** Basic characteristics of the participants from both study groups. The values are expressed as means ± standard deviations.

	WBC	DS
Number of participants	15	10
Age (years)	27.5 ± 3.1	26.2 ± 4.6
Body height (cm)	179.9 ± 7.0	180.4 ± 7.2
Body weight (kg)	71.6 ± 3.6	82.2 ± 13.6
Body mass index (BMI, kg/m^2^)	23.1 ± 1.3	27.0 ± 6.4
Body surface are (BSA, m^2^)	1.9 ± 0.1	2.0 ± 0.2
Smokers	6.67%	20%

**Table 2:** The values of oxidative stress parameters in the blood of healthy men 20 min after the whole-body cryostimulation (WBC) procedure and the dry sauna (DS) bath. The results are expressed as means ± standard deviations.

	WBC		DS	
	Baseline	After WBC	Baseline	After DS
TBARSpl [nmol MDA/mL]	0.57 ± 0.08	0.59 ± 0.08	0.34 ± 0.05^aaa^	0.35 ± 0.06^bbb^
TBARSer [nmol MDA/g Hb]	36.6 ± 13.2	41.1 ± 17.8	18.2 ± 4.1^aaa^	17.6 ± 5.0^bbb^
SOD [U/g Hb]	791.1 ± 192.5	1034.5 ± 112.6^aaa^	773.2 ± 124.3	880.2 ± 121.3^bbc^
CAT [10^4^ IU/g Hb]	74.6 ± 20.6	67.9 ± 11.2	59.8 ± 16.3	66.2 ± 16.0^cc^
GPx [U/g Hb]	17.8 ± 9.7	25.0 ± 11.5^aa^	5.9 ± 1.9^aaa^	7.4 ± 3.3^bbb^

^a^Difference versus WBC baseline value (^aa^ *P* < 0.01, ^aaa^ *P* < 0.001); ^b^difference versus post-WBC value (^bb^ *P* < 0.01, ^bbb^ *P* < 0.001); ^c^difference versus DS baseline value (^c^ *P* < 0.05, ^cc^ *P* < 0.01). TBARSer, thiobarbituric acid reactive substances in erythrocytes; TBARSpl, thiobarbituric acid reactive substances in blood plasma; SOD, superoxide dismutase; CAT, catalase; GPx, glutathione peroxidase.

## References

[1] Avery S. V. (2011). Molecular targets of oxidative stress. *Biochemical Journal*.

[2] Blagojevic D. P., Grubor-Lajsic G. N., Spasic M. B. (2011). Cold defence responses: the role of oxidative stress. *Frontiers in Bioscience*.

[3] Woźniak A. (2003). Signs of oxidative stress after exercise. *Biology of Sport*.

[4] Mila-Kierzenkowska C., Woźniak A., Szpinda M. (2012). Effects of thermal stress on the activity of selected lysosomal enzymes in blood of experienced and novice winter swimmers. *Scandinavian Journal of Clinical and Laboratory Investigation*.

[5] Sutkowy P., Woźniak A., Mila-Kierzenkowska C., Jurecka A. (2011). The concentration of thiobarbituric acid reactive substances (TBARS) and vitamins A and E in blood of amateur athletes after single whole-body cryostimulation. *Polish Journal of Sport Medicine*.

[6] Sutkowy P., Woźniak A., Olszewska-Słonina D. The changes of oxidant-antioxidant profile in the blood of healthy men after single dry sauna procedure—preliminary study.

[7] Wozniak A., Mila-Kierzenkowska C., Szpinda M., Chwalbinska-Moneta J., Augustynska B., Jurecka A. (2013). Whole-body cryostimulation and oxidative stress in rowers: the preliminary results. *Archives of Medical Science*.

[8] Wozniak A., Wozniak B., Drewa G., Mila-Kierzenkowska C. (2007). The effect of whole-body cryostimulation on the prooxidant-Antioxidant balance in blood of elite kayakers after training. *European Journal of Applied Physiology*.

[9] Kukkonen-Harjula K., Kauppinen K. (2006). Health effects and risks of sauna bathing. *International Journal of Circumpolar Health*.

[10] Livingston R. (2010). Medical risks and benefits of the sweat lodge. *Journal of Alternative and Complementary Medicine*.

[11] Zinchuk V. V., Zhadko D. D. (2012). The effect of a sauna on blood oxygen transport and the prooxidant-antioxidant balance in untrained subjects. *Human Physiology*.

[12] Prystupa T., Wołyńska A., Ślȩyński J. (2009). The effects of finish sauna on hemodynamics of the circulatory system in men and women. *Journal of Human Kinetics*.

[13] Scoon G. S. M., Hopkins W. G., Mayhew S., Cotter J. D. (2007). Effect of post-exercise sauna bathing on the endurance performance of competitive male runners. *Journal of Science and Medicine in Sport*.

[14] Buege J. A., Aust S. D., Fleisher S., Packer I. (1978). Microsomal lipid peroxidation. *Methods in Enzymology*.

[15] Esterbauer H., Cheeseman K. H., Packer L., Glazer A. N. (1990). Determination of aldehydic lipid peroxidation products: malondialdehyde and 4-hydroksynonenal. *Methods in Enzymology*.

[16] Beers R. F., Sizer I. W. (1952). A spectrophotometric method for measuring the breakdown of hydrogen peroxide by catalase. *The Journal of Biological Chemistry*.

[17] Paglia D. E., Valentine W. N. (1967). Studies on the quantitative and qualitative characterization of erythrocyte glutathione peroxidase. *The Journal of Laboratory and Clinical Medicine*.

[18] Misra H. P., Fridovich I. (1972). The role of superoxide anion in the autoxidation of epinephrine and a simple assay for superoxide dismutase. *The Journal of Biological Chemistry*.

[19] Lubkowska A., Chudecka M., Klimek A., Szyguła Z., Fraczek B. (2008). Acute effect of a single whole-body cryostimulation on prooxidant-antioxidant balance in blood of healthy, young men. *Journal of Thermal Biology*.

[20] Selfe J., Alexander J., Costello J. T. (2014). The effect of three different (−135°C) whole body cryotherapy exposure durations on elite rugby league players. *PLoS ONE*.

[21] Woźniak A., Drewa G., Woźniak B. (2005). Effect of cryogenic temperatures and exercise on lipid peroxidation in kayakers. *Biology of Sport*.

[22] Dugué B., Smolander J., Westerlund T. (2005). Acute and long-term effects of winter swimming and whole-body cryotherapy on plasma antioxidative capacity in healthy women. *Scandinavian Journal of Clinical and Laboratory Investigation*.

[23] Miller E., Saluk J., Morel A., Wachowicz B. (2013). Long-term effects of whole body cryostimulation on uric acid concentration in plasma of secondary progressive multiple sclerosis patients. *Scandinavian Journal of Clinical & Laboratory Investigation*.

[24] Sutkowy P., Woźniak A., Boraczyński T., Mila-Kierzenkowska C., Boraczyński M. (2014). The effect of a single Finnish sauna bath after aerobic exercise on the oxidative status in healthy men. *Scandinavian Journal of Clinical and Laboratory Investigation*.

[25] Pilch W., Szyguła Z., Tyka A. K. (2014). Disturbances in pro-oxidant-antioxidant balance after passive body overheating and after exercise in elevated ambient temperatures in athletes and untrained men. *PLoS ONE*.

